# Geographical Differentiation of the Daurian Ground Squirrel (*Spermophilus dauricus*) Population Based on Morphological Traits

**DOI:** 10.3390/ani15233403

**Published:** 2025-11-25

**Authors:** Xi Chen, Zhenshan Liu, Zixuan Wang, Xiaohan Liu, Ming Yang, Yu Zhou

**Affiliations:** College of Life Sciences, Shenyang Normal University, Shenyang 110034, China; xxchen0619@163.com (X.C.); lzhenshan1929@163.com (Z.L.); zizixuann@163.com (Z.W.); 15840262070@163.com (X.L.)

**Keywords:** Daurian ground squirrel, geographical populations, subspecies classification, morphological variation, multivariate analysis

## Abstract

The Daurian ground squirrel (*Spermophilus dauricus*) is an important animal model that is frequently used by researchers studying the physiological ecology of hibernation in China. There has been considerable controversy regarding the classification of its subspecies, and there is a lack of relevant comparisons of morphometric data. To clarify this issue, this study aims to systematically compare five external morphological traits and 20 cranial traits of *S. dauricus* collected from 10 locations within China to assess the phenotypic differentiation of *S. dauricus* within China. Through morphological cluster analysis (MCA), discriminant function analysis (DFA), and principal component analysis (PCA), the samples from 10 locations (FK, *n* = 5; JZ, *n* = 11; DQ, *n* = 7; KP, *n* = 6; HEB, *n* = 4; EE, *n* = 10; CF, *n* = 3; ZB, *n* = 6; CD, *n* = 8; WL, *n* = 7) can be divided into three geographical populations: the Northeast population (NE); the Inner Mongolia population (IM); and the Hebei population (HB). There is significant morphological variation in four external traits and 19 cranial traits among populations within the species. Specifically, the NE population presented greater body length, tail length, and cranial dimensions. The IM population has the shortest tail, whereas the HB population has the smallest overall body size—its tail length, however, falls between those of the NE and IM populations. These morphological differences may be related to differences in habitat. This study revealed a significant correlation between environmental factors and the morphological traits of *S. dauricus*. Redundancy analysis (RDA) further revealed that the mean annual temperature (MAT) and mean annual precipitation (MAP) significantly affected the morphological traits of *S. dauricus*. This study challenges the current subspecies classification, clarifies the geographical morphological variation pattern of *S. dauricus*, reveals the influence of the environment on the differentiation of its morphological traits, and provides new morphological evidence for resolving the controversy over its subspecies classification.

## Simple Summary

The Daurian ground squirrel (*Spermophilus dauricus*) is a key research species in the field of hibernation physiological ecology in China. However, its subspecies classification remains a subject of debate to date. One key reason is likely the lack of systematic morphological data and associated analyses. To clarify the morphological differentiation among its populations, we conducted a systematic morphological analysis of *S. dauricus* from 10 sampling sites across China. Morphological data indicate that these 10 populations have differentiated into three major geographical groups: the Inner Mongolia population, the Hebei population, and the Northeast population. Additionally, we found that mean annual temperature and mean annual precipitation significantly influence the morphological differentiation of these populations. This study clarifies the pattern of morphological variation in *S. dauricus* and provides new evidence for resolving the long-standing controversy surrounding its subspecies classification.

## 1. Introduction

External morphology and cranial traits are important criteria for classifying small mammals [1]. The Daurian ground squirrel (*Spermophilus dauricus*, Brandt, 1844) is widely distributed in Mongolia, Russia, regions north of the Yellow River, and Northeast China. Owing to its seasonal body fat accumulation and hibernation behavior, it has become a key animal model in physiological ecology studies in China. However, there has long been controversy over the subspecific classification of *S. dauricus*, and researchers often confuse it with its close relatives. This is largely due to a lack of systematic research on the variation in its morphological traits. Currently, four subspecies are generally recognized [2]: *S. d. dauricus* occurs in Russia and Mongolia, although some scholars argue that its distribution extends into China, ranging from southeast Inner Mongolia to the Greater Khingan Mountains [3]. The remaining three subspecies—*S. d. ramosus*, *S. d. mongolicus*, and *S. d. obscurus*—are endemic to China.

The ground squirrels in Beijing were described as *S. d. mongolicus* Milne-Edwards, 1867. This subspecies was once classified under *Citellus dauricus* Allen, 1940 [4], who also noted that it should be conspecific with *C. mongolicus umbratus* Thomas, 1908 [5]. Those in Jilin were described as *C. m. ramosus* Thomas, 1909. However, Bannikov considered mongolicus and ramosus forms to be the same subspecies, recognizing only two subspecies: *C. d. dauricus* and *C. d. mongolicus*. In contrast, Gromov recognized only *C. d. ramosus*. Additionally, another subspecies, *C. d. yamashinai* Kuroda, 1939, was recorded with a type locality in northern Manzhouli, Inner Mongolia, China [6], but this form was not mentioned in some of the most complete species descriptions [7,8].

Chinese scholars also hold divergent views on its subspecific classification. A comprehensive review of Chinese mammals revealed three subspecies of *S. dauricus* within China: *S. d. ramosus*, distributed in Heilongjiang, Jilin, Liaoning, eastern Inner Mongolia, and Shanxi; *S. d. mongolicus*, distributed in Beijing, Hebei, Tianjin, Henan and Shandong; and *S. d. obscurus*, distributed in northwestern Gansu and Xinjiang [2]. However, the China Biodiversity Red List states that *S. dauricus* occurs in areas north of Hebei, Inner Mongolia, and Northeast China, with no mention of its presence in northwestern China [9]. Another study classified ground squirrels in northwestern regions as Pallid ground squirrels (*S. pallidicauda*) rather than *S. dauricus* [10]. Given these inconsistencies, this study focuses on whether geographic populations in northeastern Inner Mongolia, the Northeast Plain, and Hebei exhibit subspecific and morphological differentiation; thus, no samples were collected from Gansu, Xinjiang, or other regions. Notably, authoritative global mammal monographs do not recognize subspecies of *S. dauricus*, treating all proposed subspecies as synonyms of “dauricus” [11].

On the basis of molecular evidence, Russian scholars have hypothesized that the *S. dauricus* population distributed in China should be divided into three populations: the Inner Mongolia population, the Northeast population and the Hebei population [3]. They also suggested conducting extensive sampling within China to verify this hypothesis. Although scholars have different views on the subspecific classification of *S. dauricus*, no existing studies have conducted systematic morphological measurements and statistical analyses of *S. dauricus* in different geographical populations. Given the above situation, this study aims to clarify the phenotypic differentiation characteristics of different geographical populations of *S. dauricus* through systematic analysis of morphological data, with the aim of filling the gap in the morphological data of this species’ subspecific classification and providing support for further clarifying the subspecific classification relationships of this species.

## 2. Materials and Methods

### 2.1. Animal Collection and Grouping

**Figure 1:**
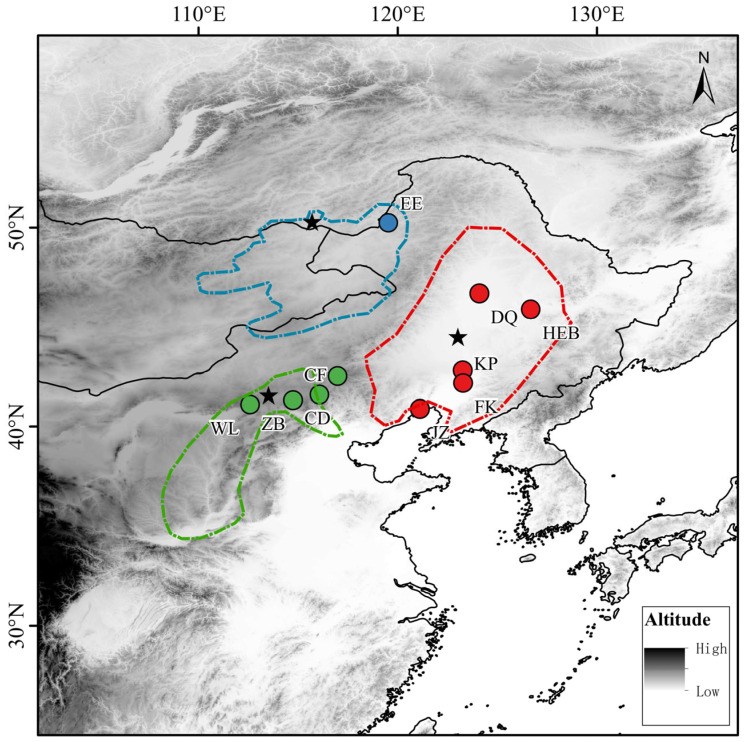
Sampling sites of *S. dauricus*. The different colors of the sampling sites represent different geographical populations. The green sampling sites represent the HB population, the red sampling sites represent the NE population, and the blue sampling sites represent the IM population. Different colored areas represent the distribution ranges of different subspecies: the red area corresponds to *S. d. ramosus*, the green area to *S. d. dauricus mongolicus*, and the blue area to *S. d. dauricus*. Asterisks indicate the type localities of the respective subspecies.

From May to September 2024, we collected 67 *S. dauricus from* 10 locations within China via cage trapping and water flooding methods [12]. We presented all the subgroups in line with the three geographical populations/subpopulations reported by Kapustina [3], namely the Northeast population (NE), the Inner Mongolia population (IM), and the Hebei population (HB) (Figure 1), and provided the latitude, longitude, and sample size for each corresponding subgroup (Table 1). After capture, the animals were quarantined and brought back to the laboratory. They were euthanized successively from October to November of the same year, and their skulls and taxidermy samples were measured and prepared. All the samples were stored in the Laboratory of Physiological Ecology at Shenyang Normal University. All procedures were licensed under the Animal Care and Use Committee of Life Science, Shenyang Normal University.

### 2.2. Morphological Trait Determination

**Figure 2:**
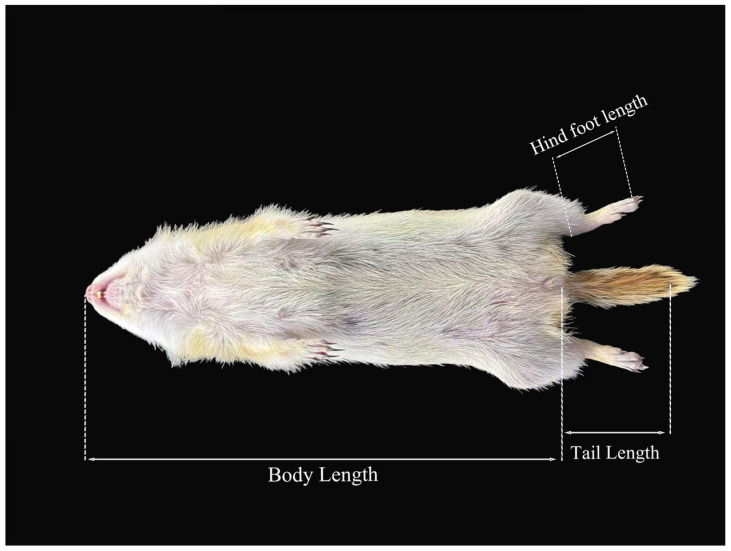
Measurement Chart of external morphological Traits for *S. dauricus*. (Due to perspective limitations, the measurement position of EH cannot be clearly visualized, so it is not marked in the figure).

**Figure 3:**
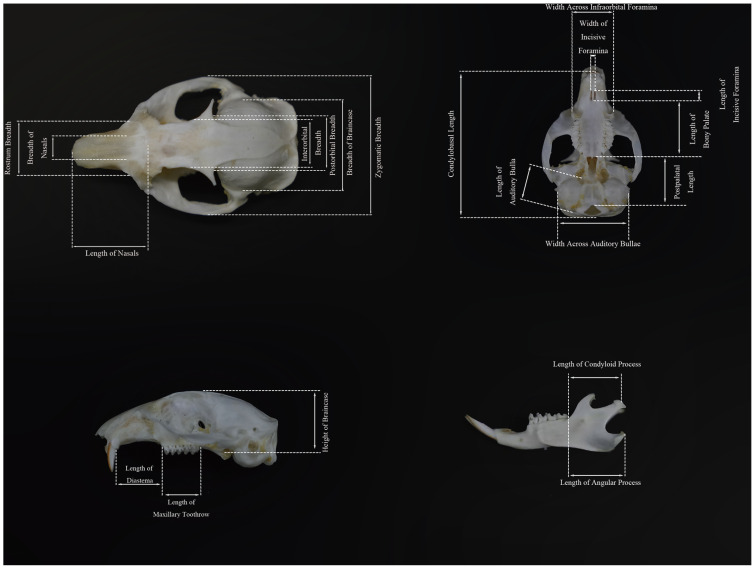
Measurement Chart of Cranial Traits for *S. dauricus*. CBL = condylobasal length. ZB = Zygomatic width. HBC = Height of braincase. RB = Rostral breadth. LN = Length of nasals. WN = Width of nasals. IOB = Interorbital breadth. LD = Length of diastema. LAB = Length of auditory bulla. LBP = Length of bony palate. PPL = Postpalatal length. BBC = Breadth of braincase. POB = Postorbital breadth. LIF = Length of incisive foramina. WIF = Width of incisive foramina. WAIF = Width across infraorbital foramen. WAB = Width of auditory bulla. LMTR = Length of maxillary toothrow. LAP = Length of angular process. LCP = Length of the condyloid process.

The morphological characteristics were measured via a digital vernier caliper (with an accuracy of 0.1 mm). Five external morphological traits and 20 cranial traits were measured for each sample [13,14]. The external morphological traits included body length (BL): Straight-line distance from the nasal apex to the anus, tail length (TL): Straight-line distance from the anus to the tail tip, excluding the terminal tail hairs, hindfoot length (HFL): Straight-line distance from the heel to the tip of the longest toe, excluding the claw, and ear height (EH): Maximum distance from the lower margin to the upper margin of the ear pinna; the tail–body ratio (T/B) was subsequently calculated. The cranial traits included the condylobasal length (CBL): Distance from the posterior margin of the occipital condyle to the anterior end of the incisor, zygomatic width (ZB): Maximum width between the lateral margins of the left and right zygomatic arches, breadth of braincase (BBC): Maximum breadth of the posterior part of the skull (neurocranium), typically at the level of the mastoid processes, height of braincase (HBC): Maximum height from the vertex of the neurocranium to the inferior margin of the auditory bulla, rostral breadth (RB): Minimum width of the anterior part of the maxilla at the rostral region, length of nasals (LN): Maximum length from the anterior margin to the posterior margin of the nostril, width of nasals (WN): Maximum width between the lateral margins of the nostril, interorbital breadth (IOB): Minimum distance between the two orbits (the distance between the depressions of the frontal bones at the posterior part of the orbits), postorbital breadth (POB): Minimum breadth of the skull at the posterior margin of the orbits, Length of diastema (LD): Distance from the posterior margin of the alveolus of the last incisor to the anterior margin of the alveolus of the ipsilateral first premolar, length of incisive foramina (LIF): Maximum length from the anterior margin to the posterior margin of the incisive foramen, width of incisive foramina (WIF): Maximum width between the lateral margins of the incisive foramen, width across infraorbital foramen (WAIF): Distance between the lateral margins of the left and right infraorbital foramina, length of bony palate (LBP): Distance from the anterior margin to the posterior margin of the palatine bone, postpalatal length (PPL): Distance from the posterior margin of the palatine bone to the anterior margin of the foramen magnum, length of auditory bulla (LAB): Maximum length from the anterior margin to the posterior margin of the auditory bulla, width of auditory bulla (WAB): Maximum width between the lateral margins of the auditory bulla, length of maxillary toothrow (LMTR): Maximum length from the anterior margin of the alveolus of the maxillary premolar to the posterior margin of the alveolus of the ipsilateral last maxillary molar, length of angular process (LAP): Straight-line distance from the tip of the mandibular angular process to the junction of the angular process base and the mandibular body, and length of the condyloid process (LCP): Straight-line distance from the highest point of the mandibular condyloid process to the junction of the condyloid process base and the mandibular ramus (Figure 2 and Figure 3).

### 2.3. Environmental Variable Data

To assess the influence of environmental factors on the morphological variations among different populations, we selected six key environmental variables: longitude, latitude, elevation, mean annual temperature (MAT), mean annual precipitation (MAP), and normalized difference vegetation index (NDVI). Environmental data were sourced from authoritative databases to ensure reliability: elevation, MAT, and MAP data were retrieved from WorldClim (https://worldclim.org/, accessed on 14 September 2025) [15]. NDVI data were obtained from the NASA LPDAAC collection in the MODIS database (https://lpdaac.usgs.gov, accessed on 14 September 2025). The MAP, MAT, Elevation and NDVI data are presented in Table S1.

### 2.4. Statistical Analysis

The data were first assessed for normality and homogeneity of variance via the Kolmogorov-Smirnov test (K-S test) and Levene’s test, respectively, via SPSS software (version 25.0). For morphological difference analysis between males and females, data that conformed to a normal distribution were analyzed via an independent-samples *t* test, whereas nonnormally distributed data were analyzed via the Mann-Whitney U test. Sexual size dimorphism (SSD) for each morphological variable was quantified via the following formula: SSD = [(male mean/female mean) − 1] × 100 [16].

If no significant morphological differences were detected between males and females, the two groups were pooled to maximize the sample size. All analyses were conducted in R (version 4.3.2), with methods selected on the basis of data compliance with statistical assumptions. When the data met both the normality and homogeneity of variance assumptions, one-way analysis of variance (ANOVA) was used to test for overall intergroup differences, followed by Tukey’s honest significant difference (HSD) post hoc test for pairwise comparisons. When the data met the normality assumption but violated homogeneity of variance, Welch’s ANOVA was applied, supplemented by the Games–Howell post hoc test. When the data violated both assumptions, the Kruskal-Wallis H test (a nonparametric equivalent of one-way ANOVA) was used, followed by Dunn’s post hoc test with Bonferroni correction to control the family-wise error rate. All the numerical results are presented as the mean ± standard error (mean ± SE). Differences were considered statistically significant when *p* < 0.05.

Specific R(4.3.2) packages were employed for distinct analytical tasks: basic parametric and nonparametric tests: “stats” package (for one-way ANOVA and Kruskal-Wallis H test); Welch’s ANOVA: “one-way tests” package; post hoc tests: “multcomp” package (Tukey’s HSD), “rstatix” package (Games-Howell test), and “FSA” package (Dunn’s test with Bonferroni correction); cluster analysis: “pheatmap” package, which implements hierarchical clustering based on Euclidean distance to quantify similarities in morphological traits among samples—this clustering reveals phenotypic affinities between samples; principal component analysis (PCA): “FactoMineR” package, which is used for dimensionality reduction in morphological datasets and extraction of key trait gradients; discriminant function analysis (DFA): “MASS” package, which is employed to build discriminant models for distinguishing between populations on the basis of morphological traits; correlation analysis: “Hmisc” package, which is used to calculate Pearson correlation coefficients between morphological variables and environment factors; redundancy analysis (RDA): “vegan” package, which quantifies the explanatory power of environment factors on morphological variation, with model significance validated via permutation tests.

## 3. Results

### 3.1. Basic Statistics

A systematic analysis was conducted on the morphological characteristics of 5 external variables and 20 cranial variables in 10 populations. The K-S test results indicated that the measured values of 14 morphological traits were normally distributed (*p* > 0.05), and 10 of them met the homogeneity of variance criterion (*p* > 0.05). The analysis of differences between males and females revealed that only one morphological variable, the length of the hind limbs, was significantly different (*p* < 0.05), and the sex bimodal index of all the morphological traits was low (SSD < 5%) (Table 2). However, this difference was biologically negligible when evaluated by the SSD index. The SSD is used to analyze differences in body size with respect to animal sex [17]. An SSD < 5% is generally recognized as indicating weak sexual dimorphism in morphological traits, as such a small magnitude of difference does not reflect meaningful biological divergence between sexes [18]. Therefore, we believe that there are no obvious sex differences in the morphological characteristics of *S. dauricus*. The subsequent analysis combines the sample sizes of males and females for comparison.

### 3.2. Morphological Clustering Analysis (MCA)

The subsequent analysis combines the sample sizes of males and females for comparison. The clustering heatmap results revealed that the body size and cranial dimensions of individuals in the KP, DQ, JZ, and FK subgroups were greater than those of individuals in the other subgroups. Additionally, the clustering results indicated that all the subgroups could be divided into two main clusters: the first cluster included the DQ, JZ, and FK subgroups; the second cluster was further subdivided into two subclusters, one composed of the ZB, CD, and WL subgroups; and the other composed of the EE, CF, and HEB subgroups (Figure S1). The clustering results are not consistent with the supposed affiliations of the subgroups to specific geographical populations as reported in existing studies.

### 3.3. Analysis of Morphological Differences Among Subgroups

For inter-subgroup difference comparisons among the 10 subgroups, two indicators (WN, LAP) had *p* < 0.05 in the preliminary statistical test, suggesting potential intergroup differences. However, when pairwise comparisons were performed between all groups (after multiple comparison correction), no statistically significant differences were observed between any groups. Given that multiple comparison correction in statistical analyses better controls Type I errors, this study ultimately adopted the corrected pairwise comparison results as the basis for conclusions. Statistical analyses revealed the following results. In terms of external morphology: For BL, the DQ (225.57 ± 2.34 mm) and FK (222.80 ± 3.81 mm) subgroups presented the highest values, whereas the ZB subgroup (194.50 ± 5.37 mm) presented the lowest values (H_9,57_ = 34.13, *p* < 0.01). For HFL, the FK (37.60 ± 0.51 mm) and JZ (37.91 ± 0.56 mm) subgroups were larger, whereas the CF (34.33 ± 0.67 mm) and CD (34.50 ± 1.18 mm) subgroups were smaller (H_9,57_ = 26.77, *p* < 0.01). The FK subgroup (66.40 ± 3.25 mm) presented the longest TL, whereas the EE (51.60 ± 1.18 mm) and HEB (51.00 ± 1.41 mm) subgroups presented the shortest TL (F_9,57_ = 3.746, *p* < 0.01). For T/B was highest in the ZB subgroup (0.31 ± 0.02), which differed significantly from the EE subgroup and other subgroups; T/B values were relatively similar among the remaining subgroups (F_9,57_ = 2.492, *p* < 0.05). There were no statistically significant differences in EH among the subgroups (H_9,57_ = 34.13, *p* = 0.1).

In cranial morphology: For CBL, the FK (45.43 ± 0.41 mm) and DQ (45.65 ± 0.72 mm) subgroups presented the highest values, whereas the EE subgroup (41.13 ± 0.30 mm) presented the lowest (F_9,57_ = 16.356, *p* < 0.01). For ZB, the FK subgroup (30.81 ± 0.45 mm) had the highest value, and the WL subgroup (27.00 ± 0.32 mm) had the lowest value (F_9,57_ = 14.02, *p* < 0.01). For RB, the JZ (9.97 ± 0.06 mm) and DQ (9.96 ± 0.32 mm) subgroups presented the longest values, whereas the EE (8.96 ± 0.10 mm) and ZB (8.95 ± 0.16 mm) subgroups presented the shortest values (F_9,57_ = 6.71, *p* < 0.01). For LIF, the FK subgroup (3.58 ± 0.06 mm) had the longest value, whereas the DQ (2.71 ± 0.21 mm) and HEB (2.79 ± 0.12 mm) subgroups had the shortest values (H_9,57_ = 32.90, *p* < 0.01).

Overall, the NE population (including the FK, DQ, and JZ subgroups) was larger in both external and cranial morphology and significantly larger than the other subgroups in 16 morphological indicators: HBC, BBC, RB, IOB, WAIF, WIF, LAB, WAB, CBL, ZB, LN, LD, LBP, PPL, LMTR, and LCP. In contrast, the HB and IM populations (the HB population, including the WL and ZB subgroups, and the IM population, including the EE subgroups) were relatively smaller. For these 16 indicators, the statistical results were as follows (respectively, ANOVA/Kruskal-Wallis: F_9,57_ = 4.72, *p* < 0.01; H_9,57_ = 30.08, *p* < 0.01; F_9,57_ = 6.71, *p* < 0.01; F_9,57_ = 5.49, *p* < 0.01; H_9,57_ = 26.65, *p* < 0.01; H_9,57_ = 24.11, *p* < 0.01; F_9,57_ = 4.96, *p* < 0.01; H_9,57_ = 41.71, *p* < 0.01; F_9,57_ = 16.36, *p* < 0.01; F_9,57_ = 14.02, *p* < 0.01; F_9,57_ = 13.51, *p* < 0.01; F_9,57_ = 8.59, *p* < 0.01; F_9,57_ = 9.84, *p* < 0.01; F_9,57_ = 6.88, *p* < 0.01; H_9,57_ = 32.57, *p* < 0.01; F_9,57_ = 6.39, *p* < 0.01) (Table S2). However, the BL and TL values were greater in the NE population, indicating that the animals in the NE population had relatively greater body sizes. The HB population has the smallest body length, whereas the IM population has the shortest tail.

### 3.4. Principal Components Analysis (PCA) Based on Morphological Traits

PCA was conducted on 25 morphological indicators of *S. dauricus* at the subgroup level. The analysis generated three principal components, which collectively accounted for 58.40% of the phenotypic variation. Among these, the first principal component (PC1) exhibited the greatest variation, with an eigenvalue of 10.1 and a variance contribution rate of 40.42% (Table 3). This dominant component alone accounted for nearly half of the total explained variation, highlighting its critical role in capturing the most substantial phenotypic differentiation among subgroups. Specifically, PC1 had high positive loadings concentrated on cranial indicators such as CBL (0.942), ZB (0.897), LBP (0.865), PPL (0.818), LN (0.813), and LD (0.811) reflecting the overall variation in cranial morphology. The second principal component (PC2, eigenvalue = 2.7, variance contribution = 10.68%) was strongly correlated with tail-related traits like T/B (0.896) and TL (0.752), indicating variation in body proportions. The third principal component (PC3, eigenvalue = 1.8, variance contribution = 7.3%) was associated mainly with LAP (0.458) and WAB (0.633), revealing potential differences in diet and sensory system specialization among the subgroups.

**Figure 4:**
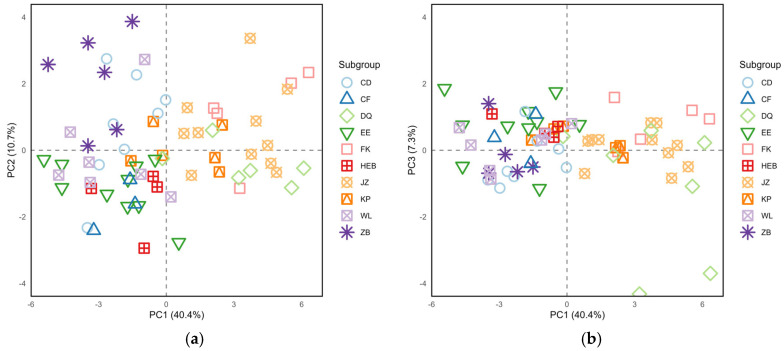
(**a**) Plots of principal component factors 1 and 2 for the morphological data of different populations of *S. dauricus*; (**b**) Plots of principal component factors 1 and 3 for the morphological data of different populations of *S. dauricus*.

The results of the PC1-PC2 scatter plot revealed that individuals from the JZ, FK, and DQ subgroups were distributed mainly on the right side of the PC1 axis, suggesting that these individuals likely had relatively larger cranial sizes. Individuals from the ZB, CD, and WL subgroups were located on the left side of the PC1 axis, whereas those from the EE, HEB, and CF subgroups were distributed in the lower-left quadrant. Notably, individuals in the KP subgroup were widely distributed across all four quadrants, indicating substantial morphological variation (Figure 4a). In the PC1-PC3 scatter plot, individuals from the JZ, FK, and DQ subgroups still aggregated mainly on the right side of the PC2 axis, whereas individuals from the other subgroups were concentrated on the left side (Figure 4b).

### 3.5. Clustering Based on Discriminant Function Analysis (DFA) of Morphological Traits

**Figure 5:**
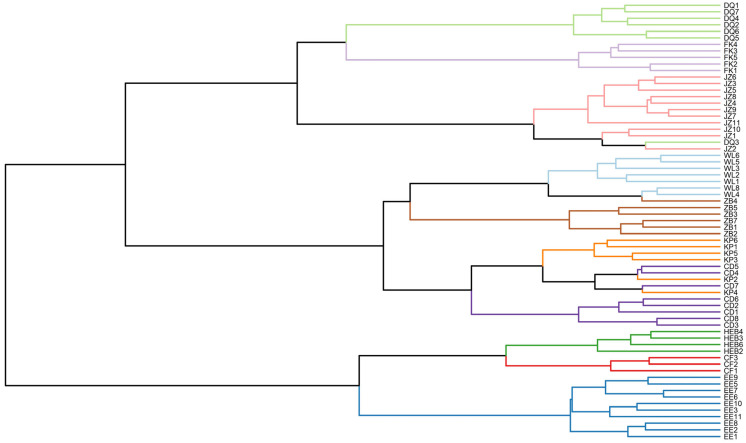
Clustering analysis diagram based on the morphological data.

The dendrogram structure from the cluster analysis (based on the DFA results) clearly shows two main branching patterns (Figure 5). First, the EE, CF, and HEB subgroups clustered into an independent main branch, suggesting close morphological affinity among these three subgroups. Second, the remaining subgroups clustered into a second main branch, which was further subdivided into two sub branches: the DQ, FK, and JZ subgroups clustered into the first sub branch, whereas the WL, ZB, CD, and KP subgroups clustered into the second sub branch.

On the basis of branch proximity in the clustering dendrogram, we reclassified the 10 subgroups into three major geographical populations: specifically, the NE population included the DQ, FK, and JZ subgroups; the HB population included the WL, ZB, CD, and KP subgroups; and the IM population included the EE, CF, and HEB subgroups.

### 3.6. Correlation Analysis Between Morphological Traits and Environmental Factors

**Figure 6:**
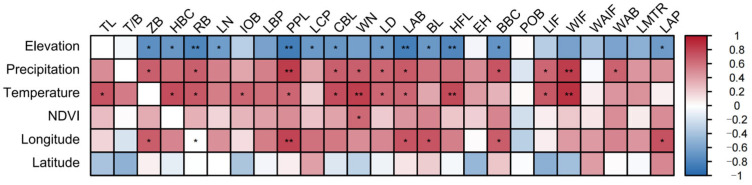
Correlation analysis between environmental factors and morphological traits of *S. dauricus*, * *p* < 0.05 and ** *p* < 0.01.

This study further examined the correlations between six key environmental factors—latitude, longitude, elevation, MAT, MAT, and NDVI—and multiple morphological traits of *S. dauricus*, with key results visualized in a heatmap (Figure 6).

From the overall trend of these correlations, different environmental factors exhibited distinct differences in their influences on morphological traits. Specifically, elevation, MAP, MAT, and longitude were significantly correlated with most morphological traits (*p* < 0.05), whereas latitude and the NDVI had relatively limited effects on these traits.

As revealed by the correlation heatmap, elevation was significantly negatively correlated with 12 morphological traits (ZB, HBC, RB, LN, PPL, LCP, CBL, LD, BL, HFL, BBC, and LAP) (*p* < 0.05); annual precipitation was significantly positively correlated with 11 traits (ZB, RB, PPL, CBL, WN, LD, LAB, BBC, LIF, WIF, and WAB) (*p* < 0.05); and the annual average temperature was significantly positively correlated with 12 traits (TL, HBC, RB, IOB, PPL, CBL, WN, LD, LAB, HFL, LIF, and WIF) (*p* < 0.05).

Notably, the NDVI was significantly positively correlated with only WN and was not significantly correlated with the other morphological traits. In contrast, longitude was significantly positively correlated with seven traits (ZB, RB, PPL, LAB, BL, BBC, and LAP) (*p* < 0.05), whereas latitude was not significantly correlated with any of the measured morphological traits.

### 3.7. Redundancy Analysis (RDA) of Morphological Traits and Environmental Factors

This study employed RDA to explore the relationships among populations, morphological traits, and environmental factors. The eigenvalues of the first two RDA axes were 6.66 and 1.45, respectively. These two axes (RDA1 and RDA2) accounted for 67.6% and 15.7% of the total variance, respectively, with a cumulative explanatory power of 83.3% (Table 4).

**Figure 7:**
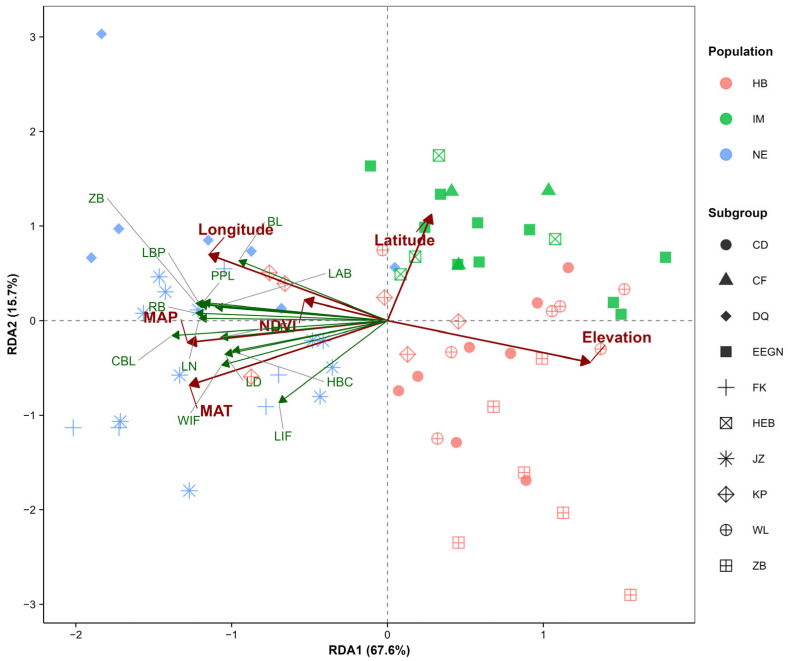
Redundancy analysis of *S. dauricus* morphological traits and environmental factors. Redundancy analysis (RDA) of *S. dauricus* populations, illustrating the associations between morphological traits and environmental factors. Different symbols denote distinct populations, and different colors represent different DFA groups. The arrows indicate environmental factors, with their length and direction reflecting the extent and trend of influence on population morphological traits.

The results from the RDA ordination plot indicated that the first ordination axis (RDA1) was positively correlated with latitude and elevation, whereas the second ordination axis (RDA2) was negatively correlated with longitude, MAT, NDVI, and MAP. Furthermore, all the morphological characteristics presented positive correlations with longitude, MAT, NDVI, and MAP and negative correlations with latitude and elevation (Figure 7). MAT, MAP, and elevation had extremely significant effects on the morphological variation in *S. dauricus* (*p* < 0.01), whereas longitude had a significant effect (*p* < 0.05); however, latitude and the NDVI had no statistically significant effect on the variation (*p* > 0.05) (Table 5). Notably, these findings were largely consistent with the results of the correlation analysis conducted in this study.

## 4. Discussion

In mammals, intraspecific geographical variation in morphology is a widespread phenomenon, often exhibiting complex patterns, including clinal variation, discrete types, and mosaic types [19]. External and cranial morphological data play irreplaceable roles in exploring intraspecific phylogenetic relationships [20,21,22]. Nadler investigated differences between two subspecies of *Spermophilus richardsonii* via cranial data and reported that the skull size of *S. r. richardsonii* was significantly larger than that of *S. r. elegans* [23]. Through morphometric analyses, Gunduz demonstrated significant morphological differences among three ground squirrel species in Turkey [24]. Sinitsa confirmed the taxonomic status of the extinct ground squirrel *Spermophilus citelloides* by comparing morphological traits among *Spermophilus* species [25].

In this study, we conducted systematic morphological analyses to explore the geographical variation in *S. dauricus*. By integrating this variation with recorded subspecific classifications and performing multivariate analyses, we propose a revised classification of Chinese *S. dauricus* into three geographical populations. Specifically, we suggest adjusting the traditional classification of Northeast subspecies (originally including the CF, EE, HEB, DQ, FK, KP, and JZ subgroups) and the Hebei subspecies (originally including the WL, ZB, and CD subgroups). However, whether these three geographical populations have differentiated at the subspecies level requires further genetic analysis combined with genomic data. Morphological clustering and DFA-based clustering adopt distinct analytical logics: the former is predicated on the species’ existing three-subspecies classification, conducting confirmatory analysis of 10 small populations. Its core objective is to verify whether these small populations conform to the morphological aggregation pattern of the predefined subspecies, with grouping based on the comprehensive similarity of all morphological traits and their congruence with the subspecies classification. The latter does not predefine any subspecies groups, instead directly performing exploratory analysis on the 10 small populations. By weighting key morphological traits with significant intergroup differences and low intragroup variation, it automatically identifies natural clustering patterns, thereby revealing the intrinsic morphological differentiation among the small populations. Despite their differing analytical logics, the results of the two clustering methods are highly consistent, both classifying *S. dauricus* within China into three geographical populations: (1) the NE population, which includes the FK, DQ, and JZ subgroups and corresponds to *S. d. ramosus*; (2) the HB population, which includes the ZB, CD, WL, and KP subgroups and corresponds to *S. d. mongolicus*; and (3) the IM population, which includes the EE, HEB, and CF subgroups and corresponds to *S. d. dauricus*. The results of morphometric analyses, heatmaps, and PCA clearly revealed that the NE population was significantly larger than the other two populations in terms of 15 morphological traits. This morphological differentiation may be closely related to the habitat, survival conditions, and hibernation characteristics of *S. dauricus*.

On the basis of these factors, individuals in the NE geographical population are relatively larger in size. Moreover, the morphological trait data from these measurements can serve as important diagnostic criteria for differentiating *S. dauricus* populations across regions. After visualizing the data via multivariate analyses, we found that the three geographical populations had distinct distribution centers in the PCA ordination plot. The clustering results generated from the DFA outputs for these 10 populations indicate that they can be divided into two main branches: one including the JZ, DQ, and FK populations and the other consisting of two additional groups (WL, CD, ZB, KP; and EE, HEB, CF). These clustering results were highly congruent with the PCA results. Additionally, this study documented the numerical ranges and mean values of key external and cranial measurements for each geographical population. These traits are the primary drivers of differences in body size and cranial morphology among the three geographical populations, and the data provide robust support for the subspecific classification of *S. dauricus*.

We further explored the associations between environmental factors and the morphological differentiation of different geographical populations of *S. dauricus*. In current animal ecology research, the academic community has developed two classic explanatory frameworks for how environmental factors regulate the development of animal body size and the evolution of skull dimensions—Bergmann’s law and Allen’s rule. Bergmann’s Law states that in regions with high ambient temperatures, animals exhibit smaller body sizes and larger surface areas; in contrast, the opposite pattern occurs in regions with lower environmental temperatures [26]. In contrast, Allen’s rule emphasizes that in cold environments, protruding structures on the body surface of endothermic animals tend to shorten to reduce surface area and conserve heat; in warm environments, these structures tend to be relatively elongated to facilitate heat dissipation [27].

Studies on other ground squirrel species have confirmed that temperature and precipitation directly or indirectly regulate body size differentiation. For example, increased precipitation enhances plant productivity, providing more abundant food resources for Mexican ground squirrels (*S. mexicanus*), which indirectly promotes larger body size in this species [28]. Similarly, Arctic ground squirrels (*Urocitellus parryii plesius*) exhibit body size differences between forest and meadow habitats, a pattern indirectly mediated by temperature and precipitation [29].

Our correlation analysis revealed that most morphological traits of *S. dauricus* were significantly positively correlated with annual mean temperature and annual mean precipitation and significantly negatively correlated with latitude and elevation. Specifically, the EE population, which is distributed in the northernmost region of China, has smaller TL and T/B values, whereas populations in Northeast China (JZ, FK, and DQ) have larger values for these traits. This pattern aligns closely with the core prediction of Allen’s law. RDA further supported these findings: MAP and MAT collectively account for the largest proportion of phenotypic variation in *S. dauricus*, with MAP exhibiting the strongest statistical association and explaining the highest share of this variation. Additionally, population clusters identified via factor analysis show distinct aggregation in the ordination space, indicating that long-term environmental adaptation has driven morphological differentiation among geographical populations.

In the Northeast China Plain, the natural grassland habitat of the NE population has been largely replaced by farmland, shifting the dominant habitat type from natural grassland to agricultural land. This transformation has altered food resources from herbaceous plants to hard-shelled crops, such as peanuts, which are more concentrated in distribution and nutritionally richer. The larger skulls and body sizes in the NE population accommodate more developed masticatory muscles, facilitating efficient mastication of these hard-shelled crops. Furthermore, human agricultural activities have disrupted the foraging behavior of natural predators, reducing selection pressure on auditory vigilance; notably, the NE population exhibits lower auditory specialization than the IM and HB populations.

Hibernation differences have further exacerbated morphological divergence among geographical populations. The NE population inhabits the warm, humid Northeast Plain and emerges from hibernation earlier, typically ending hibernation in March [30]. By this time, plant resources have already begun to grow, ensuring timely food availability. This earlier emergence provides individuals with a longer growth period and sufficient time to acquire nutrients, promoting the development of larger body sizes. In contrast, the HB population occupies high-elevation grasslands with cold climates, and the IM population resides in high-latitude, high-elevation regions. These areas experience seasonal fluctuations in food resources and greater predation pressure from natural predators. Both geographical populations enter hibernation earlier (late September) and emerge later (March–May) [31,32]. Compared with the IM and HB populations, the NE population of *S. dauricus* experiences lower survival pressure, benefits from a longer growth period, and inhabits an environment with less seasonal fluctuations in food resources and more favorable habitat conditions (elevation, MAT, and MAP), ultimately resulting in a larger body size.

Notably, this study has certain limitations in classifying populations of S. dauricus in northeastern Inner Mongolia, China, as it relies solely on data from populations within China. Therefore, it is necessary to integrate the morphological data of *S. dauricus* from Mongolia and Russia and analyze the morphological affinity between these transboundary populations and those in northeastern Inner Mongolia, China—thereby verifying the morphological affinity between the IM population and *S. d. dauricus*. Additionally, molecular biological methods can be used to detect genetic differentiation among populations, further verifying whether these morphological differences have reached the level of subspecies differentiation. Moreover, more in-depth experimental investigations can be conducted on the interaction mechanism between key environmental factors and morphological traits to reveal the underlying physiological and ecological processes. These studies provide a more comprehensive explanation for the relationship between the subspecies differentiation pattern and the ecological adaptation of *S. dauricus*.

## 5. Conclusions

This study conducted systematic measurements and multivariate statistical analyses of five external morphological traits and 20 cranial morphological traits of the *S. dauricus* populations at 10 sampling sites within China. It clarified the morphological differentiation characteristics of the populations and their environmental driving mechanisms.

The results showed that there were significant morphological differences among different geographical populations of *S. dauricus*, and this differentiation could support the classification of these populations into three geographical groups: NE, IM, and HB.

This grouping better reflects the adaptive morphological responses of the populations to regional environmental conditions and is inconsistent with the existing subspecies classification system for this species [2]. Mean Annual Precipitation (MAP), Mean Annual Temperature (MAT), and altitude are the key environmental factors influencing the species’ morphological differentiation. These factors jointly shape variations in body size and cranial morphology of *S. dauricus*. The NE population inhabits the warm and humid Northeast Plain, where food resources are more abundant, the hibernation period is shorter, and predation pressure is lower. Consequently, it has evolved a longer tail, larger body size, and relatively less specialized auditory structures. In contrast, the HB population (distributed at higher altitudes) and the IM population (distributed at higher latitudes) occupy habitats with significantly lower annual average temperatures, dominated by arid grasslands. These habitats have relatively scarce food resources and longer hibernation periods, leading the two populations to evolve a smaller body size, shorter tail, and relatively more developed auditory structures. This study not only clarified the morphological variation patterns of the geographical populations of *S. dauricus* but also provided new morphological evidence to resolve the subspecies classification disputes, laying a foundation for subsequent studies on the species’ population evolution, ecological adaptation, and taxonomic revision.

## Figures and Tables

**Table 1:** Population and sampling information of *S. dauricus*.

Population	Subgroup	Longitude	Latitude	Total Sample Size
NE ^1^	FK	123.30056	42.16323	5
	KP	123.26325	42.77108	6
	JZ	121.11992	40.87936	11
	DQ	123.86672	46.77926	7
	HEB	126.70408	45.87715	4
IM ^2^	EE	119.54838	50.21693	10
HB ^3^	CD	116.07503	41.59463	8
	ZB	114.74765	41.29196	6
	CF	116.99673	42.5387	3
	WL	112.59453	41.08083	7

^1^ NE = Northeast population. ^2^ IM = Inner Mongolia population. ^3^ HB = Hebei population. (reference Kapustina et al., 2018 [3]).

**Table 2:** Sexual differences in the morphological indices of *S. dauricus* (the measurement data are presented as the means ± standard errors, sample sizes, and results of *t* tests/U tests).

Morphological Traits	Female (*n* = 24)	Male (*n* = 43)	*t* Test/Mann-Whitney U Test		
			T/U	Sig. (2-Tailed)	SSD (%)
BL ^1^	210.790 ± 2.830	213.670 ± 1.853	437	0.301	1.3
HFL ^2^	37.080 ± 0.288	35.790 ± 0.358	337	0.018	3.6
EH ^3^	7.813 ± 0.199	7.477 ± 0.105	416	0.157	4.5
TL ^4^	59.250 ± 1.942	57.330 ± 1.041	0.873	0.388	3.3
T/B ^5^	0.282 ± 0.011	0.270 ± 0.005	1.022	0.314	4.4
CBL ^6^	43.652 ± 0.481	42.927 ± 0.268	1.317	0.196	1.7
ZB ^7^	29.017 ± 0.374	28.451 ± 0.179	1.367	0.181	2.0
HBC ^8^	15.168 ± 0.135	14.980 ± 0.129	0.946	0.348	1.3
RB ^9^	9.473 ± 0.136	9.313 ± 0.081	1.076	0.286	1.7
LN ^10^	16.773 ± 0.299	16.578 ± 0.135	0.593	0.557	1.2
WN ^11^	4.823 ± 0.118	4.761 ± 0.068	0.489	0.626	1.3
IOB ^12^	8.725 ± 0.126	8.464 ± 0.118	1.886	0.064	3.0
LD ^13^	12.193 ± 0.194	11.807 ± 0.105	1.747	0.089	3.3
LAB ^14^	8.909 ± 0.100	8.743 ± 0.061	1.506	0.137	1.9
LBP ^15^	17.978 ± 0.196	17.727 ± 0.112	1.202	0.234	1.4
PPL ^16^	15.293 ± 0.171	15.107 ± 0.100	1.001	0.321	1.2
BBC ^17^	20.270 ± 0.195	19.925 ± 0.272	471	0.556	1.7
POB ^18^	11.722 ± 0.135	11.641 ± 0.090	488	0.714	0.7
LIF ^19^	3.078 ± 0.081	3.021 ± 0.062	467	0.517	1.9
WIF ^20^	1.720 ± 0.063	1.753 ± 0.044	480	0.638	1.9
WAIF ^21^	9.948 ± 0.299	9.617 ± 0.130	462	0.476	3.4
WAB ^22^	21.423 ± 0.220	21.232 ± 0.315	500	0.829	0.9
LMTR ^23^	9.593 ± 0.089	9.795 ± 0.079	419	0.205	2.1
LAP ^24^	11.994 ± 0.180	11.687 ± 0.120	419	0.202	2.6
LCP ^25^	10.635 ± 0.223	10.338 ± 0.112	1.326	0.189	2.9

^1^ BL = Body length. ^2^ HFL = Hindfoot length. ^3^ EH = Ear height. ^4^ TL = Tail length. ^5^ T/B = Tail–body ratio. ^6^ CBL = condylobasal length. ^7^ ZB = Zygomatic width. ^8^ HBC = Height of braincase. ^9^ RB = Rostral breadth. ^10^ LN = Length of nasals. ^11^ WN = Width of nasals. ^12^ IOB = Interorbital breadth. ^13^ LD = Length of diastema. ^14^ LAB = Length of auditory bulla. ^15^ LBP = Length of bony palate. ^16^ PPL = Postpalatal length. ^17^ BBC = Breadth of braincase. ^18^ POB = Postorbital breadth. ^19^ LIF = Length of incisive foramina. ^20^ WIF = Width of incisive foramina. ^21^ WAIF = Width across infraorbital foramen. ^22^ WAB = Width of auditory bulla. ^23^ LMTR = Length of maxillary toothrow. ^24^ LAP = Length of angular process. ^25^ LCP = Length of the condyloid process.

**Table 3:** Factor loadings, eigenvalues, percentage of total variance explained and cumulative variance explained by the first three principal components for the principal component analysis of the morphological data of *S. dauricus*.

Variables	PC1	PC2	PC3
TL ^1^	0.503	0.752	0.028
TB ^2^	0.199	0.896	−0.003
ZB ^3^	0.897	−0.12	−0.009
HBC ^4^	0.62	0.134	−0.216
RB ^5^	0.762	−0.106	0.046
LN ^6^	0.813	0.083	0.141
IOB ^7^	0.717	0.134	−0.314
LBP ^8^	0.865	−0.086	−0.244
PPL ^9^	0.818	−0.084	−0.059
LCP ^10^	0.641	−0.231	0.198
CBL ^11^	0.942	0.006	−0.158
WN ^12^	0.604	−0.078	−0.25
LD ^13^	0.811	0.062	−0.107
LAB ^14^	0.76	−0.129	−0.073
BL ^15^	0.608	−0.569	−0.013
HFL ^16^	0.669	0.184	0.109
EH ^17^	0.129	0.515	−0.051
BBC ^18^	0.506	−0.128	0.651
POB ^19^	0.198	0.042	−0.254
LIF ^20^	0.349	0.424	0.369
WIF ^21^	0.502	0.338	0.1
WAIF ^22^	0.458	−0.339	−0.471
WAB ^23^	0.633	−0.156	0.558
LMTR ^24^	0.447	0.074	−0.221
LAP ^25^	0.489	−0.241	0.458
Eigenvalues	10.1	2.7	1.8
Variance explained (%)	40.4	10.7	7.3
Cumulative variance explained (%)	40.4	51.1	58.4

^1^ TL = Tail length. ^2^ T/B = Tail–body ratio. ^3^ ZB = Zygomatic width. ^4^ HBC = Height of braincase. ^5^ RB = Rostral breadth. ^6^ LN = Length of nasals. ^7^ IOB = Interorbital breadth. ^8^ LBP = Length of bony palate. ^9^ PPL = Postpalatal length. ^10^ LCP = Length of the condyloid process. ^11^ CBL = condylobasal length. ^12^ WN = Width of nasals. ^13^ LD = Length of diastema. ^14^ LAB = Length of auditory bulla. ^15^ BL = Body length. ^16^ HFL = Hindfoot length. ^17^ EH = Ear height. ^18^ BBC = Breadth of braincase. ^19^ POB = Postorbital breadth. ^20^ LIF = Length of incisive foramina. ^21^ WIF = Width of incisive foramina. ^22^ WAIF = Width across infraorbital foramen. ^23^ WAB = Width of auditory bulla. ^24^ LMTR = Length of maxillary toothrow. ^25^ LAP = Length of angular process.

**Table 4:** Factor loadings, eigenvalues, percentage of total variance explained, and cumulative variance explained for the first two axes obtained from the RDA of the morphological data of *S. dauricus*.

Variables	RDA1	RDA2
TL ^1^	−0.459	−0.442
TB ^2^	−0.153	−0.567
ZB ^3^	−0.821	0.086
HBC ^4^	−0.652	−0.237
RB ^5^	−0.809	0.001
LN ^6^	−0.804	−0.015
IOB ^7^	−0.514	−0.315
LBP ^8^	−0.787	0.047
PPL ^9^	−0.791	0.093
LCP ^10^	−0.551	0.396
CBL ^11^	−0.9	−0.163
WN ^12^	−0.599	−0.122
LD ^13^	−0.688	−0.25
LAB ^14^	−0.733	0.059
BL ^15^	−0.643	0.374
HFL ^16^	−0.706	−0.146
EH ^17^	−0.028	−0.382
BBC ^18^	−0.456	0.281
POB ^19^	−0.08	−0.054
LIF ^20^	−0.438	−0.582
WIF ^21^	−0.682	−0.353
WAIF ^22^	−0.258	0.299
WAB ^23^	−0.592	0.172
LMTR ^24^	−0.62	0.112
LAP ^25^	−0.319	0.489
Eigenvalues	6.66	1.45
Variance explained (%)	67.6	15.7
Cumulative variance explained (%)	67.6	83.3

^1^ TL = Tail length. ^2^ T/B = Tail–body ratio. ^3^ ZB = Zygomatic width. ^4^ HBC = Height of braincase. ^5^ RB = Rostral breadth. ^6^ LN = Length of nasals. ^7^ IOB = Interorbital breadth. ^8^ LBP = Length of bony palate. ^9^ PPL = Postpalatal length. ^10^ LCP = Length of the condyloid process. ^11^ CBL = condylobasal length. ^12^ WN = Width of nasals. ^13^ LD = Length of diastema. ^14^ LAB = Length of auditory bulla. ^15^ BL = Body length. ^16^ HFL = Hindfoot length. ^17^ EH = Ear height. ^18^ BBC = Breadth of braincase. ^19^ POB = Postorbital breadth. ^20^ LIF = Length of incisive foramina. ^21^ WIF = Width of incisive foramina. ^22^ WAIF = Width across infraorbital foramen. ^23^ WAB = Width of auditory bulla. ^24^ LMTR = Length of maxillary toothrow. ^25^ LAP = Length of angular process.

**Table 5:** Factor loadings, F values, and significance of the environmental variables in the RDA.

Environment Variable	RDA1	RDA2	F	P
MAP ^1^	−0.847	−0.195	18.26	0.001
MAT ^2^	−0.809	−0.521	4.23	0.003
Elevation	0.902	−0.283	7.71	0.001
NDVI ^3^	−0.332	0.079	1.52	0.133
Longitude	−0.802	0.452	1.44	0.185
Latitude	0.117	0.81	2.45	0.038

^1^ MAP = Mean annual precipitation. ^2^ MAT = Mean annual temperature. ^3^ NDVI = Normalized difference vegetation index.

## Data Availability

All original contributions from this study are included in this article. Additional information is available from the corresponding author upon request.

## Supplementary Materials

The following supporting information can be downloaded at: https://www.mdpi.com/article/10.3390/ani15233403/s1, Figure S1: Cluster analysis heatmap of morphological traits; Table S1: Climate, Elevation, and NDVI Information for Subgroups of *S. dauricus*; Table S2: Results of external and cranial morphological measurements of *S. dauricus*.
