# Supplementary material for: Geographical Differentiation of the Daurian Ground Squirrel (Spermophilus dauricus) Population Based on Morphological Traits
Source: Animals (Basel). 2025 Nov 25;15(23):3403. doi: 10.3390/ani15233403 (PMC12691073; doi:10.3390/ani15233403)
Supplement: Supplementary file 1 [file animals-15-03403-s001.zip › animals-3985797-supplementary.pdf]

Table S1. Population and sampling information of *S. dauricus*.

Subgroup	MAP(mm)[1]	MAT(℃)[2]	Elevation(m)	NDVI[3]
FK	50.08	7.65	54	0.39
KP	44.75	7.51	92	0.45
JZ	47.17	8.99	32	0.30
DQ	34.92	4.32	138	0.37
HEB	43.25	4.17	115	0.37
EE	31.08	-2.21	558	0.27
CD	36.00	2.33	1546	0.37
ZB	30.50	4.50	1357	0.26
CF	34.17	1.14	1499	0.31
WL	30.33	2.07	1865	0.37

[1] MAP = Mean annual precipitation. [2] MAT = Mean annual temperature. [3] NDVI = Normalized difference vegetation index.

Table S2. Results of external and cranial morphological measurements of *S. dauricus.*

Traits	FK	KP	JZ	DQ	HEB	EE	CD	ZB	CF	WL	F/H	P
**BL**[1]	222.80±3.81^{cd}	218.00±4.24^{acd}	217.09±2.18^{acd}	225.57±2.34^{c}	216.25±3.82^{abcd}	208.50±4.03^{abd}	204.50±4.40^{ab}	194.50±5.37^{b}	218.33±1.33^{acd}	207.14±3.32^{abd}	H=34.13	<0.01
**HFL**[2]	37.60±0.51	37.33±0.33	37.91±0.56	37.57±0.57	35.25±0.63	35.50±0.54	34.50±1.18	36.17±0.60	34.33±0.67	35.00±0.44	H=26.77	<0.01
**TL**[3]	66.40±3.25^{a}	59.67±1.93^{abc}	61.64±2.01^{ab}	62.14±3.65^{abc}	51.00±1.41^{bc}	51.60±1.18^{c}	58.63±2.77^{abc}	60.00±2.18^{abc}	50.00±3.61^{bc}	55.00±3.27^{abc}	F=3.746	<0.01
**EH**[4]	8.40±0.37	7.50±0.22	7.64±0.21	7.21±0.38	7.50±0.29	7.20±0.13	8.31±0.42	7.75±0.31	7.17±0.17	7.29±0.18	H=14.57	0.10
**T/B**[5]	0.30±0.02^{ab}	0.28±0.01^{ab}	0.28±0.01^{ab}	0.28±0.02^{ab}	0.24±0.01^{ab}	0.25±0.01^{a}	0.28±0.02^{ab}	0.31±0.02^{b}	0.23±0.02^{ab}	0.27±0.02^{ab}	F=2.492	0.02
**HBC**[6]	16.06±0.24^{a}	15.32±0.41^{abc}	15.37±0.14^{abd}	15.66±0.24^{ab}	14.79±0.36^{abc}	14.56±0.16^{c}	14.47±0.21^{c}	15.20±0.24^{abc}	14.45±0.28^{bc}	14.60±0.31^{bc}	F=4.72	<0.01
**BBC**[7]	21.46±0.39^{d}	20.38±0.34^{abd}	20.64±0.21^{ad}	20.40±0.17^{abcd}	20.35±0.08^{abcd}	20.22±0.24^{abcd}	19.92±0.14^{abc}	17.72±1.66^{c}	19.64±0.13^{abc}	19.39±0.26^{bc}	H=30.08	<0.01
**RB**[8]	9.65±0.24^{ab}	9.45±0.13^{ab}	9.97±0.06^{a}	9.96±0.32^{a}	9.40±0.16^{ab}	8.96±0.10^{b}	9.04±0.13^{b}	8.95±0.16^{b}	9.13±0.17^{ab}	8.99±0.20^{b}	F=6.71	<0.01
**IOB**[9]	8.69±0.22^{abcd}	8.61±0.21^{abcd}	9.04±0.11^{a}	9.02±0.22^{ab}	8.02±0.11^{cd}	8.03±0.15^{c}	8.74±0.14^{abd}	8.37±0.18^{abcd}	8.07±0.16^{bcd}	8.43±0.14^{abcd}	F=5.49	<0.01
**POB**[10]	11.46±0.38	11.60±0.25	12.02±0.12	11.83±0.21	10.63±0.42	11.91±0.13	11.76±0.11	11.55±0.31	11.24±0.35	11.60±0.17	H=14.82	0.10
**WAIF**[11]	9.86±0.28^{ac}	9.15±0.08^{b}	9.15±0.08^{b}	11.84±0.97^{c}	9.67±0.08^{abc}	9.62±0.08^{abc}	9.33±0.11^{ab}	9.37±0.10^{abc}	9.28±0.09^{ab}	9.40±0.11^{ab}	H=26.65	<0.01
**LIF**[12]	3.58±0.06^{b}	3.13±0.09^{abc}	3.38±0.09^{bc}	2.71±0.21^{a}	2.79±0.12^{a}	2.85±0.05^{a}	2.91±0.18^{a}	3.16±0.08^{abc}	2.77±0.06^{a}	2.95±0.13^{ac}	H=32.90	<0.01
**WIF**[13]	1.94±0.09^{b}	1.76±0.06^{ab}	2.11±0.12^{b}	1.72±0.08^{ab}	1.73±0.10^{ab}	1.51±0.06^{a}	1.64±0.04^{ab}	1.63±0.09^{ab}	1.59±0.11^{ab}	1.64±0.06^{ab}	H=24.11	<0.01
**LAB**[14]	9.12±0.19^{ab}	8.87±0.24^{ab}	9.14±0.09^{a}	9.20±0.18^{ab}	8.88±0.09^{ab}	8.61±0.10^{b}	8.51±0.11^{b}	8.39±0.12^{b}	8.45±0.03^{b}	8.66±0.07^{b}	F=4.96	<0.01
**WAB**[15]	22.68±0.35^{c}	22.00±0.31^{ac}	22.54±0.20^{c}	22.03±0.38^{ac}	20.57±0.31^{ab}	20.94±0.22^{ab}	20.68±0.23^{ab}	18.77±1.82^{b}	21.51±0.28^{abc}	20.77±0.21^{ab}	H=41.71	<0.01
**CBL**[16]	45.43±0.41^{a}	43.61±0.37^{abc}	45.12±0.41^{a}	45.65±0.72^{ab}	42.30±0.61^{abcd}	41.13±0.30^{d}	42.48±0.25^{bcd}	41.91±0.23^{cd}	41.99±0.31^{cd}	41.59±0.43^{cd}	F=16.356	<0.01
**ZB**[17]	30.81±0.45^{c}	28.70±0.23^{ab}	29.54±0.23^{ac}	30.54±0.49^{abd}	28.43±0.24^{bd}	27.75±0.37^{bd}	28.13±0.17^{bd}	27.39±0.29^{bd}	28.41±0.09^{abd}	27.00±0.32^{d}	F=14.02	<0.01

**LN[18]**	18.19±0.22[c]	16.65±0.21[ab]	17.58±0.16[ac]	17.98±0.31[c]	15.65±0.33[bd]	16.28±0.27[bd]	15.43±0.25[d]	15.94±0.35[bd]	16.34±0.51[abd]	16.01±0.10[bd]	F=13.51	＜0.01
**WN[19]**	4.95±0.15	5.21±0.17	5.09±0.15	5.02±0.14	4.59±0.20	4.42±0.22	4.69±0.07	4.53±0.07	4.53±0.35	4.65±0.14	F=2.74	0.01
**LD[20]**	13.26±0.38[ab]	11.57±0.13[ac]	12.58±0.18[b]	12.60±0.28[ab]	11.78±0.32[abc]	11.46±0.18[ac]	11.61±0.12[ac]	11.63±0.22[abc]	11.08±0.10[c]	11.48±0.20[ac]	F=8.59	＜0.01
**LBP[21]**	18.37±0.18[bcd]	18.03±0.29[abcd]	18.46±0.18[bc]	18.99±0.29[c]	17.44±0.30[abd]	17.12±0.15[a]	17.39±0.11[ad]	17.11±0.25[a]	17.80±0.15[abcd]	17.37±0.19[ad]	F=9.84	＜0.01
**PPL[22]**	16.02±0.22[a]	15.39±0.09[ab]	15.69±0.21[a]	15.72±0.26[a]	15.30±0.48[ab]	14.82±0.13[b]	15.03±0.14[ab]	14.39±0.16[b]	14.82±0.29[ab]	14.45±0.14[b]	F=6.88	＜0.01
**LMTR[23]**	9.78±0.18[abcd]	10.00±0.15[abd]	10.10±0.10[d]	10.02±0.09[ad]	9.51±0.13[abcd]	9.54±0.23[bc]	9.47±0.11[abc]	9.55±0.11[abcd]	10.10±0.09[abd]	9.21±0.18[c]	H=32.57	＜0.01
**LAP[24]**	12.35±0.30	12.06±0.22	11.67±0.22	12.29±0.26	12.23±0.21	12.06±0.20	11.29±0.17	10.76±0.48	11.96±0.31	11.65±0.39	H=18.72	0.03
**LCP[25]**	10.45±0.35[abc]	10.22±0.24[abcd]	11.15±0.15[bc]	11.41±0.40[c]	10.43±0.05[abcd]	10.60±0.24[abc]	10.14±0.22[abd]	9.10±0.27[d]	9.91±0.34[abcd]	10.08±0.17[ab]	F=6.39	＜0.01

[a,b,c,d]Means in a row not sharing a common letter are significantly different ($p < 0.05$). [1] BL = Body length. [2] HFL = Hindfoot length. [3] EH = Ear height. [4] TL = Tail length. [5] T/B = Tail–body ratio. [6] HBC = Height of braincase. [7] BBC = Breadth of braincase. [8] RB = Rostral breadth. [9] POB = Postorbital breadth. [10] IOB = Interorbital breadth. [11] WAIF = Width across infraorbital foramen. [12] LIF = Length of incisive foramina. [13] WIF = Width of incisive foramina. [14] LAB = Length of auditory bulla. [15] WAB = Width of auditory bulla. [16] CBL = condylobasal length. [17] ZB = Zygomatic width. [18] LN = Length of nasals. [19] WN = Width of nasals. [20] LD = Length of diastema. [21] LBP = Length of bony palate. [22] PPL = Postpalatal length. [23] LMTR = Length of maxillary toothrow. [24] LAP = Length of angular process. [25] LCP = Length of the condyloid process.

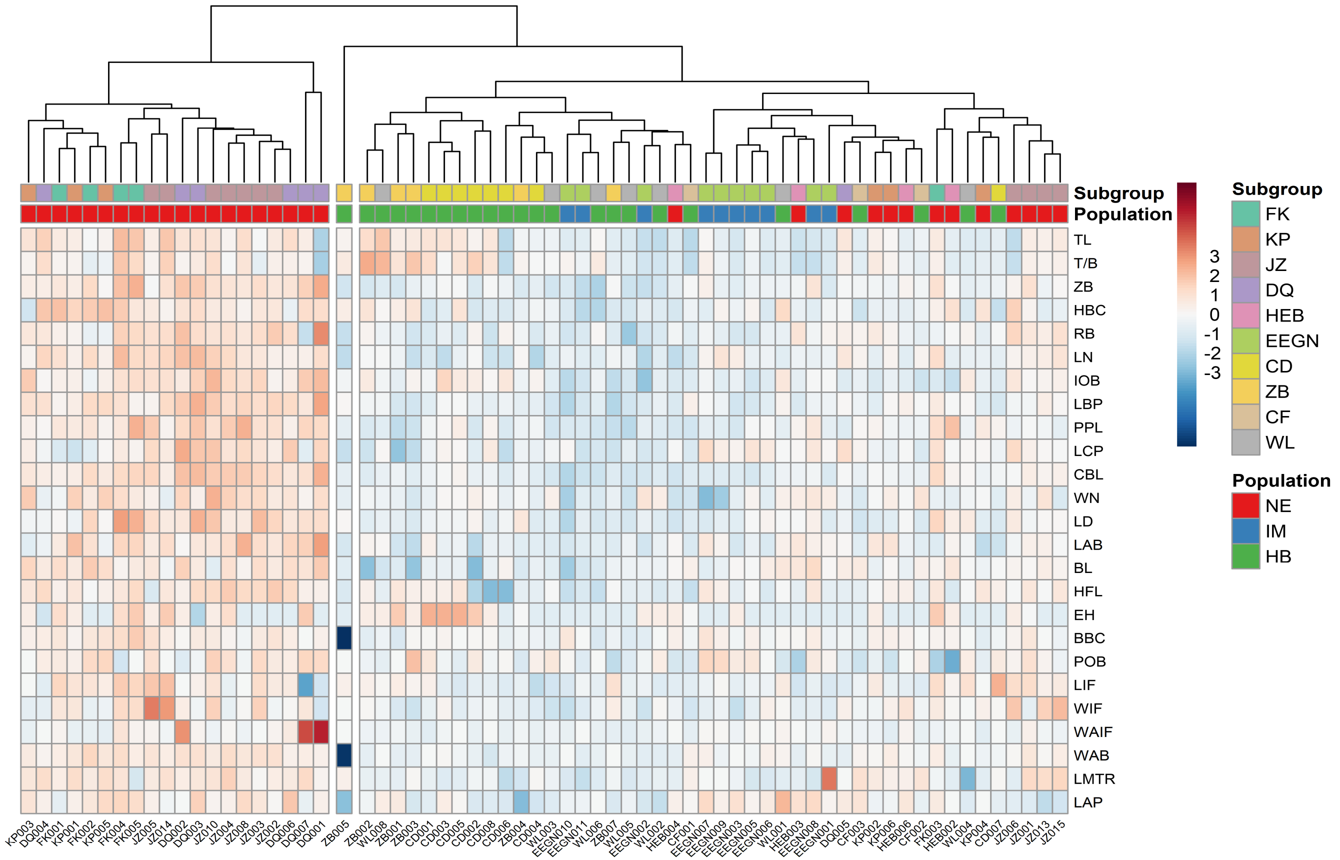

Figure S1. Cluster analysis heatmap of morphological traits. Clustering analysis of the morphological traits of different *S. dauricus* populations. Different colors in the heatmap represent varying levels of phenotypic similarity, and the dendrogram illustrates the hierarchical relationships among populations. Distinct colors also denote separate geographical populations.
